# Rehydration before Application Improves Functional Properties of Lyophilized *Lactiplantibacillus plantarum* HAC03

**DOI:** 10.3390/microorganisms9051013

**Published:** 2021-05-08

**Authors:** Karina Arellano-Ayala, Juhwan Lim, Subin Yeo, Jorge Enrique Vazquez Bucheli, Svetoslav Dimitrov Todorov, Yosep Ji, Wilhelm-Heinrich Holzapfel

**Affiliations:** 1 Department of Advanced Convergence, Handong Global University, 558 Handong-ro, Heunghae-eup, Buk-gu, Pohang-si, Gyeongbuk, Pohang 37554, Korea; karay.mx@gmail.com (K.A.-A.); jhlim@microbes.bio (J.L.); jorge_jbv@hotmail.com (J.E.V.B.); slavi310570@abv.bg (S.D.T.); 2 HEM Inc., 404, Ace Gwanggyo Tower 3, 77, Changnyong-daero 256-gil, Yeongtong-gu, Suwon-si, Gyeonggi 16229, Korea; ysji@microbes.bio; 3 HEM Inc., Business Incubator Center 103, Handong Global University, 558 Handong-ro, Heunghae-eup, Buk-gu, Pohang-si, Gyeongbuk, Pohang 37554, Korea; sbyeo@microbes.bio

**Keywords:** freeze-drying, viability, adhesion, functionality, probiotic

## Abstract

Preservation of probiotics by lyophilization is considered a method of choice for developing stable products. However, both direct consumption and reconstitution of dehydrated probiotic preparations before application “compromise” the survival and functional characteristics of the microorganisms under the stress of the upper gastro-intestinal tract. We evaluated the impact of different food additives on the viability, mucin adhesion, and zeta potential of a freeze-dried putative probiotic, *Lactiplantibacillus* (*Lp*.) *plantarum* HAC03. HAC03-compatible ingredients for the formulation of ten rehydration mixtures could be selected. Elevated efficacy was achieved by the B-active formulation, a mixture of non-protein nitrogen compounds, sugars, and salts. The survival of *Lp. plantarum* HAC03 increased by 36.36% compared rehydration with distilled water (4.92%) after passing simulated gastro-intestinal stress conditions. Cell viability determined by plate counting was confirmed by flow cytometry. B-active formulation also influenced *Lp. plantarum* HAC03 functionality by increasing its adherence to a Caco-2 cell-line and by changing the bacterial surface charge, measured as zeta potential.Hydrophobicity, mucin adhesion and immunomodulatory properties of *Lp. plantarum* HAC03 were not affected by the B-active formulation. The rehydration medium also effectively protected *Lp. plantarum* ATCC14917, *Lp. plantarum* 299v, *Latilactobacillus sakei* (*Lt.*) HAC11, *Lacticaseibacillus (Lc.) paracasei* 532, *Enterococcus faecium* 200, and *Lc. rhamnosus* BFE5263.

## 1. Introduction

Most probiotic preparations on the market are presented either as viable bacterial cells or cultures, as ingredients of fermented food products or as freeze-dried microbial preparations [1]. For the commercialization and distribution of beneficial bacterial preparations such as probiotics, it is imperative to formulate transportable, shelf-stable concentrates by which their intrinsic functional properties will be supported [2]. The long-term preservation of probiotics and other beneficial microorganisms is commonly achieved by applying different drying (dehydration) procedures, with freeze-drying (lyophilization) as the commonly preferred option for the conservation of starter cultures for applications in the food industry [3].

However, during lyophilization, the microbial cells are exposed to stress conditions such as freezing, vacuumization, cryoprotectants, and low water activity that may cause sublethal cell damage. Factors that may play a role in bacterial cell survival include intrinsic strain resistance, initial cell concentration, cell density, growth conditions, growth phase at harvesting, and the (cryo-) protective agents used [4,5,6,7]. Moreover, the effectiveness of lyophilization is directly related to the type of applied cryoprotectant. Even when considering strain-dependent variation in response to the damaging effects of freeze-drying, cryoprotectant agents such as maltodextrin (5%) and glycerol (2%) were found generally effective and economically feasible for the lyophilization of starter cultures for implementation under robust conditions such as direct inoculation for cassava fermentation [8]. A combination of skim-milk (10%, *w/v*) and sucrose (10%, *w/v*), supplemented with sodium glutamate (2.5%, *w/v*), was found to be successful for the cryoprotection of *Ligilactobacillus (Lg.) salivarius* during freeze-drying, and for yielding superior growth and survival [7]. Physiological deterioration will affect the viability and functionality of the culture and may be caused by cell shrinkage and the resulting disruption of cell integrity (by cell wall damage or as a result of membrane lipid oxidation), and by DNA, RNA and protein denaturation [4,9]. Several studies underline the importance of viable cells on probiotic functionality. In 2004, Galdeano and Perdigón [10] demonstrated that viable *Lacticaseibacillus* (*Lc.*) *casei* cells stimulated the intestinal mucosal immune system to a greater extent than non-viable bacteria in the gut of mice. Pelletier et al., in 2001, [11] also pointed out the importance of viable cells when comparing the effectiveness of viable and non- viable probiotics in the digestion of lactose in a clinical trial with lactose-intolerant subjects. In addition to viability, the functionality of probiotics may strongly be impacted by freeze-drying and preservation methods. Iaconelli et al., in 2015, [12] found that viability, immunomodulatory, and adhesion capacity of cells differ according to the drying process and the kind of bacteria, while in 2019, Kiekens et al. [13] reported that the pili of *Lc. rhamnosus* GG were sheared off during spray-drying, thereby affecting adherence of this strain.

Stress response induced by various physicochemical factors during de- and rehydration can support the restoration of cell homeostasis and thereby its vitality and performance [14]. Various cryoprotectants and even immobilization may be used for maintaining the microorganism’s viability during the freeze-drying process [15]. However, not all strains survive the process equally well and at the same rate [16], suggesting intra-species diversity in response to lyophilization-related stress conditions.

Independent of the drying method, rehydration involves an important step in the recovery of dehydrated microorganisms; even with careful procedures supporting survival during freezing, drying and storage, an inadequate rehydration step may lead to poor cell viability and a low final survival rate [17]. During the rehydration process, the cells are subjected to a rapid change in the physical–chemical conditions, usually within a few seconds, during which the cells transfer from the solid (dry) and dormant state to a hydrated colloid or suspension, thereby initiating metabolic activity in the cells. However, the viability of a freeze-dried microorganism could be compromised during and after re-hydration. Osmotic shock is a major attribute to the detrimental effect of reconstitution of a freeze-dried culture. Other factors that may also affect the viability of cells during rehydration include composition of the rehydrating solution, osmolarity, the volume ratio, re-hydration rate, time, temperature, and type of microorganism [18]. Successful rehydration is based on retaining the integrity of the cells as support for the microorganism’s functionality. On the other hand, for some microorganisms such as dried yeast, the viability, fermenting and leavening ability will probably not be significantly affected by the rehydration procedure [19]. The least possible damage to microbial cells during rehydration may be ascertained by careful selection of the matrix or excipients that incorporate the (probiotic) culture [20]. It is therefore essential to supply a suitable environment and verify the strain’s compatibility and adaptability to the reconstituting solution in order to regain its functionality [21].

In order to reach the intestinal target site in a metabolically active and functional condition, probiotic candidates should survive the passage of the upper gastro-intestinal tract (GIT) [1]. In addition, ability to adhere to mucosal surfaces and the intestinal epithelial lining is also considered an important feature of probiotic functionality, thereby conferring a competitive advantage important for its establishment in the human GIT [22]. The Food and Agriculture Organization and the World Health Organization (FAO/WHO, 2002) [19,23] jointly recommended several in vitro tests to assess the functionality of probiotic microorganisms in the human body; these include resistance to gastric acidity and the bile salts typical of the duodenum, plus adherence to mucus and/or human epithelial cells. The simulated stomach and duodenum passage assay (SSDP) mimics the main stress conditions that probiotic microorganisms experience along the upper digestive tract after ingestion and enables the prediction of the behavior and tolerance of a microbial strain [24]. Survival in and adaptation to the intestinal lumen will promote interaction with the native (autochthonous) microbiota and/or stimulation of the host immune response. This constitutes the basis of both the probiotic mode of action and the resulting beneficial effects [25].

Flow cytometry is a technique originally applied for the quantification of eukaryotic cells. Moreover, it has been adapted for the analysis of viability, metabolic state, and antigenic markers of bacteria. Its accuracy in the determination of live, dead, damaged, and total bacteria in real time emphasizes its value [26]. The use of propidium iodide, a nucleic acid-specific, red-fluorescent dye that binds to double-stranded DNA by intercalating between base pairs, allows the differentiation of live and dead bacteria. Live cells with intact membranes generally exclude the dye, while it easily penetrates the permeable membranes of non-viable cells [27].

Adhesion to intestinal epithelial cells and mucus is imperative for microbial persistence in environments such as the GIT as habitat for microorganisms [25,28], and in addition, facilitating the establishment of host–bacteria interactions [29]. Several models have been developed to assess probiotic adhesion in vitro. Some of them are based on the use of human colon adenocarcinoma cell line Caco-2 and mucus membrane proteins, such as collagen, laminin, fibronectin, and mucin. However, bacterial adhesion is a complex mechanism that involves non-specific and specific interactions such as ligand receptors. Non-specific interactions correspond to physicochemical interactions between the microorganisms’ cell surface and mucin or intestinal epithelial cells. Characteristics of the bacterial cell surface such as hydrophobicity, surface charge, and electron donor–acceptors play an important role [30]. Bacterial cell hydrophobicity is usually associated with hydrophobic surface glycoproteins or hydrophilic polysaccharides [31]. In Gram-positive bacteria, the wall teichoic acid, lipoteichoic acid, and peptidoglycan define the anionic charge of the cells [32]. Some authors refer to the zeta potential as an indication of the relative quantity of negatively or positively charged groups, the variation of which reflects the differences in the surface structures among strains [33]. It has previously been used to characterize and predict intestinal bacterial adhesion to the GIT [34].

The purpose of this study was to evaluate the effect of different food-grade additives on the survival, activity, and functionality of freeze-dried *Lp. plantarum* HAC03, a putative probiotic. The information served as a basis for composing an optimal formulation for the restoration of probiotic properties lost or diminished during freeze-drying. In vitro parameters such as survival under simulated GIT conditions, adhesion to the enterocyte-like Caco-2/TC-7 cell-line and to mucin, and immune stimulation served as indicators of rehydration effectivity and potential restoration of functionality. *Lp. plantarum* HAC03 viability in real time and bacteria cell surface changes and integrity were corroborated by flow cytometry and by measuring the zeta potential and hydrophobicity, respectively.

## 2. Materials and Methods

### 2.1. Strains and Growth Conditions

*Lp. plantarum* HAC03 with probiotic potential and previously isolated from white kimchi, Korean fermented cabbage [35], was employed as a test strain. *Lp. plantarum* subsp. *plantarum* ATCC 14917 was used as a reference strain, and *Lp. plantarum* 299v [36] as a commercial probiotic control strain. Following the standardization of the formulation, its influence on the viability and survival was also determined on *Latilactobacillus* (*Lt.*) *sakei* HAC11, *Lc. paracasei* 532, *Lp. plantarum* ATCC 14917, *E. faecium* 200, and *Lc. rhamnosus* BFE 5263. All strains were cultivated in De Man, Rogosa and Sharpe (MRS, Becton, Dickinson and Company—BD, Franklin Lakes, NJ, USA) media under anaerobic conditions (Anaerobic Chamber Whitley DG250, Don Whitley Scientific, Bingley, UK) at 37 °C for 18 h and stored at −80 °C in a Cryotube F570-86 deep freezer (Eppendorf, Hamburg, Germany) in the presence of 30% glycerol. Initially, 1% (*v/v*) of each strain was activated in 5 mL MRS broth and propagated by two sub-culturing steps in MRS broth and/or on MRS agar before each experiment. Unless otherwise specified, fresh cells were prepared by harvesting the cultures at 10,000× *g* for 5 min, washed, and re-suspended in sterile phosphate buffer saline 1X (PBS, Lonza, Basel, Switzerland) to a final concentration of 1 × 10^8^ CFU/mL.

### 2.2. Fermentation and Freeze-Drying Process

*Lp. plantarum* HAC03 was sub-cultured by 1% inoculation into 50 mL MRS broth followed by incubation under anaerobic conditions at 37 °C for 18 h. Subsequently, the culture volume was up-scaled to 4 L in a 5 L fermenter M300 (Mettler-Toledo, Columbus, OH, USA). Fermentation was conducted at 37 °C with nitrogen gas aeration and 110 rpm agitation for 17 h while maintaining the pH at 5.6 by constantly feeding in sterile 1 N HCl and/or 5 N NaOH. The cultures were collected in 500 mL sterile polypropylene bottles and harvested by centrifugation at 10,000× *g* for 20 min. The pellets were washed twice with 1X PBS, weighed, and resuspended in 10% skim-milk (BD) with 5% sucrose (Daegu Chemicals, Daegu, Korea) as cryoprotectant at a 1:2 ratio. The bacterial mix was transferred to metal plates and stored at −80 °C for 24 h in a deep freezer (Eppendorf, Hamburg, Germany). The frozen cultures were then placed in a 4.5 L benchtop freeze-dryer (Labconco Freezone, Kansas City, KS, USA) and lyophilized at a condenser temperature of −54 °C and at 0.02 psi pressure for 24 h. The dried bacteria were pulverized manually, under aseptic conditions, with a metal mortar and pestle, and stored in 50 mL polypropylene tubes at −20 °C to avoid lipid oxidation and culture deterioration.

### 2.3. Food Additives and Rehydration Conditions

Forty food-grade additives from the Food and Drug Administration (FDA) list [37] were selected for developing a new probiotic formulation for protecting *Lp. plantarum* HAC03 freeze-dried cells against osmotic shock and harsh conditions in the GIT after ingestion and to maintain or recover probiotic properties that could be compromised during freeze-drying. The selected additives included carbohydrates such as arabinose, maltose (USB, Cleveland, OH, USA), xylose, rhamnose, mannose, fructose, mannitol, glucose, trehalose, alginic acid (Sigma Aldrich, St. Louis, MO, USA), sucrose, sorbitol and starch (Daejung Chemicals, Busan, Korea), proteins and nitrogenous compounds such as albumin, pepsin (Sigma Aldrich, St. Louis, MO, USA), gelatin, peptone soy (Daejung Chemicals, Busan, Korea) and yeast extract (BD), amino acids such as L-arginine, L-ornithine, L-glutamic acid, L-proline, L-lysine (Sigma Aldrich), L-serine, L-threonine, L-aspartic acid (Georgia Chem, Atlanta, GA, USA), L-tryptophan (Kanto Chemical Co., Kanto Kagaku, Singapore), L-phenylalanine (Junsei Chemical, Tokyo, Japan) and L-tyrosine (Samchun Chemicals, Daejeon, Korea), salts such as sodium phosphate, sodium L-tartrate (Sigma Aldrich) and sodium bicarbonate (Yakuri, Kyoto, Japan), organic acids such as malic acid (Sigma Aldrich) and pyruvic acid (Daejung Chemicals), osmolytes such as betaine (Daejung Chemicals) and taurine (Sigma Aldrich) and vitamins such as riboflavin (Sigma Aldrich), thiamine hydrochloride (Daejung Chemicals) and L-ascorbic acid (TCI, Tokyo, Japan). Additive-microorganism compatibility was tested by mixing 0.01 g of bacteria powder (~1 × 10^9^ CFU/g) and the corresponding component to 0.001 M of each ingredient, or 0.02 g in the case of proteinaceous components or complex mixtures such as yeast extract, starch, and alginic acid to normalize the effect of osmotic pressure in the dried cells (Table S1). The different powder mixtures were rehydrated at 25 °C for 1 min with 1 mL autoclaved distilled water (DW) and subjected to the different essays.

### 2.4. Simulated Gastro-Intestinal Passage Resistance Assay

To evaluate the protecting effectivity of the selected 40 food additives listed before, the rehydrated *Lp. plantarum* HAC03 was exposed to the in vitro Simulated Stomach Duodenum Passage (SSDP), after which its survivability was determined under simulated physiological conditions of the human GIT. The SSDP essay was carried out as established by Haberer et al. [24] and further adapted by Ji et al. [38], with some modifications.

Freeze-dried bacteria and food additives mixtures were rehydrated as described before, and 1 mL of the mixture was subjected to simulated stomach conditions by adding 9 mL of 1X PBS pH 2.5 (pH adjusted with 1 N HCl). The obtained suspensions were vortexed, and the pH was measured and adjusted to 2.5 ± 0.2 by adding 1 N HCl or 1 N NaOH if needed. The process was conducted by incubation at 37 °C for 1 h in an anaerobic chamber (Don Whitley Scientific, Bingley, UK), directly followed by the addition of 17 mL of synthetic duodenum juice with pH 6.0 ± 0.2 (6.4 g/L NaHCO_3_, 0.239 g/L KCl and 1.28 g/L NaCl) and 4 mL of bile salts (10% ox-gall; BD) to simulate passage through the small intestine. The samples were incubated for two additional hours under the same conditions, during which assay samples were taken after 0, 1, and 3 h of incubation to determine the viability of the bacteria after exposure to acid and bile stress, by viable plate counting on MRS agar. *Lp. plantarum* HAC03 controls comprised fresh cells cultivated in MRS as mentioned before, adjusted to 1 × 10^9^ CFU/mL, while the freeze-dried bacteria were rehydrated only with DW.

### 2.5. Bacterial Adhesion to Mucin

The mucin adhesion test was performed according to the method described by Laparra and Sanz [39], with some modifications. Crude mucin type II (Sigma Aldrich) was diluted in 1X PBS (Lonza, Basel, Switzerland) to a final concentration of 5 mg/mL and loaded in aliquots of 1 mL into polycarbonate 12-well plates (SPL, Gyeonggi-do, Korea). The plates were incubated at 37 °C under anaerobic conditions, the mucin removed after 1 h, and the wells washed twice with 1X PBS (Lonza). Bacterial adhesion was determined by adding 1 mL of 1 × 10^8^ CFU/mL fresh and rehydrated freeze-dried *Lp. plantarum* HAC03 cells with or without food additives into the mucin-treated wells. The plates were incubated under anaerobic conditions, and 1 h later the cultures were removed. The wells were carefully washed twice with 1X PBS, and the mucin-attached cells were finally resuspended in 1 mL 1X PBS. The number of cells attached to mucin was calculated by viable plate counting on MRS agar in triplicate, and wells without mucin were used as controls.

### 2.6. Bacterial Zeta Potential

Changes in the surface groups of the bacteria were indirectly measured by determining the surface charge of the fresh and rehydrated freeze-dried *Lp. plantarum* HAC03 cells with or without the food additives incorporated into the mix. The assay was carried out by mixing the previously rehydrated bacteria with 9 mL of double-distilled water (DDW) at pH 2.0 to reach a final concentration of 1 × 10^8^ CFU/mL. The pH was measured and corrected to pH 2.0 by adding 0.1 N HCl or 0.1 N NaOH, and 800 μL samples were loaded in DTS1070 cuvettes. The samples were measured on a Zetasizer Nano ZEN 3600 after 2 min equilibration time (Malvern Panalytical, Malvern, UK) and the Smoluchowski model was used to convert electrophoretic mobility data to zeta potential values [34], which were considered as the average of three reads.

### 2.7. Product Formulation

Based on analyses of the initial data generated from evaluations of the effects of 40 different food grade ingredients, seven food additives were selected based on their performance for protecting *Lp. plantarum* HAC03 freeze-dried cells during the simulated GIT passage, for enhancing bacterial adhesion to mucin, and modulating zeta potential values close to fresh overnight cell cultures. Five preparations (A to E) were formulated (Table 1, with different proportions of a combination of different food additives.

Effectivity of the formulations was first evaluated based on supporting *Lp. plantarum* HAC03 viability after SSDP, using plate counting and subsequent confirmation in real time by flow cytometry. The formulation with the best outcome was also assessed by in vitro essays for cell adhesion and immune stimulation to determine whether there was an enhancement of adhesion to Caco-2/TC-7 and immune response stimulation in a macrophage cell line, which are probiotic properties frequently compromised by the freeze-drying process. The impact of the selected formulation on mucin adhesion, zeta potential and hydrophobicity of *Lp. plantarum* HAC03 were tested as well.

### 2.8. Determination of Bacterial Viability by Flow Cytometry

The effectiveness of the formulation that supported the highest *Lp. plantarum* HAC03 viability percentages in plate counting after SSDP was further confirmed in real time by flow cytometry using the dye exclusion method with propidium iodide (PI) (Sigma Aldrich) according to the protocol established by R&D systems 2016 [40], with minor modifications. The samples were subjected to SSDP as described before, however this time, the bacterial suspensions, after stomach and duodenum simulation passage, were diluted up to 1 × 10^6^ CFU/mL in 1X PBS and harvested by centrifugation at 10,000× *g* for 10 min. Supernatants were discarded and the pellets re-suspended in 1 mL of flow cytometry staining buffer composed of 1X PBS, 0.5% bovine serum albumin (BSA, Sigma Aldrich) and 0.05% NaN_3_ (Sigma Aldrich). The cells were stained by adding 20.4 μL PI to a final concentration of 30 μg/mL and incubating for 1 min in the dark after gently mixing. Bacterial viability was determined by Flow Cytometer ZE5 and Everest software v 2.2.08.0 (Bio-Rad Laboratories, Hercules, CA, USA). Fresh overnight cultures and freeze-dried *Lp. plantarum* HAC03, rehydrated only with distilled water, were used as controls.

### 2.9. Adhesion to Intestinal Epithelial Cell Line

The influence of the formulation on adherence of *Lp. plantarum* HAC03 to the human enterocyte-like Caco-2/TC-7 cell-line after freeze-drying and SSDP was also determined, according to the protocol of Botes et al. [41]. Fresh cultures of *Lp. plantarum* subsp. *plantarum* ATCC 14917 and *Lp. plantarum* 299 v were used as authentic and probiotic reference strains, respectively. *Lp. plantarum* HAC03 fresh cells were used as controls, while freeze-dried cells with and without the formulation mixture were used as experimental groups. Fresh cultures were harvested (as described before) and the obtained fresh cells, as well as the freeze-dried cells, were resuspended and/or rehydrated with DW, as described. All cultures were subjected to SSDP, and after bile stress incubation, the bacteria were harvested at 3000× *g* for 20 min, washed three times with PBS 1X, and re-suspended in 10 mL Minimal Essential Medium (MEM) cell culture media (Sigma Aldrich) supplemented with 20% Fetal Bovine Serum (FBS), 2 mM glutamine, and 1% non-essential amino acids. To assess the bacterial adhesion, 2 mL of the suspensions were incubated with 1 × 10^5^ CFU/Caco-2/TC-7 monolayer at 37 °C in a 5% CO_2_ + 95% air atmosphere for 1.5 h. Caco-2/TC-7 monolayer cells were washed three times with cold PBS to remove the non-adhering bacteria, and detached by adding 0.25% of trypsin solution (Promega, Fitchburg, WI, USA) and incubating for 15 min at 37 °C. To quantify the number of bacteria adhering to the Caco-2/TC-7 cells, the samples were serially diluted, and plate-counted on MRS agar.

### 2.10. Immune Stimulation In Vitro

The immune stimulatory properties of *Lp. plantarum* HAC03 freeze-dried cells rehydrated with the formulation was investigated in a macrophage cell line. Fresh and freeze-dried cells were prepared and subjected to SSDP, as described, and finally, cultures were washed and resuspended in DMEM media with 20% FBS to a final concentration of 1 × 10^6^ CFU/mL. The mouse macrophage cell line was grown in Dulbecco’s Modified Eagle Medium (DMEM) with 20% FBS, 2 mM glutamine, and 1% non-essential amino acids. To stimulate the macrophage, 2 mL of the bacterial suspension were incubated with 5 log CFU/macrophage monolayer for 14 to 16 h at 37 °C in a 5% CO_2_, 95% air atmosphere mixture. After incubation, the medium was carefully removed with a suction pump. The cells were washed twice with cold 1X PBS and 500 μL of Trizol were added for extracting the macrophage mRNA. A template of 200 ng RNA was used for cDNA synthesis and qRT-PCR was carried out. The primers used were IL-1b forward 5′-TCG CTC AGG GTC ACA AGA AA-3′ and reverse 5′-CAT CAG AGG CAA GGA GGA AAA C-3′ [42], MCP-1 forward 5′-GCA GTT AAC GCC CCA CTC A-3′ and reverse 5′-CCC AGC CTA CTC ATT GGG ATC A-3′ [43], TNFa forward 5′-TGG GAC AGT GAC CTG GAC TGT-3′ and reverse 5′-TTC GGA AAG CCC ATT TGA GT-3′ [42], IL-10 forward 5′-GGT TGC CAA GCC TTA TCG GA-3′ and reverse 5′-ACC TGC TCC ACT GCC TTG CT-3′ [44], TGFb forward 5′-CCC AGC ATC TGC AAA GCT C-3′ and reverse 5′-GTC AAT GTA CAG CTG CCG CA-3′ [45]. The primers ARBP forward 5′-TCA CTG TGC CAG CTC AGA AC-3′ and reverse 5′-AAT TTC AAT GGT GCC TCT GG-3′ [46] were used to normalize the transcription levels of the target genes. Quantitative real-time PCR was performed with 50 ng of cDNA and SYBR Premix Ex Taq II (Takara, Japan) using the Step-One Plus real-time PCR system (Applied Biosystems, Foster, CA, USA). The polymerase activation step at 95 °C for 30 s was followed by 40 cycles of denaturation at 95 °C for 5 s and annealing at 60 °C for 30 s. Subsequently, melting curve analysis was carried out by heating the reactions from 65 to 96 °C in 0.3 °C intervals while monitoring fluorescence. The relative gene transcription was calculated as the log_2_ of ΔΔCT.

### 2.11. Bacterial Hydrophobicity

The influence of the selected formulation on *Lp. plantarum* HAC03 hydrophobicity after rehydration was additionally investigated. Hydrophobicity and the surface charge (zeta potential) of bacterial cells play an important role in their adhesion; these features have been reported to be affected by the freeze-drying process. The assay was performed according to Arellano et al. [47], with some modifications, where the hydrophobicity was evaluated by measuring *Lp. plantarum* HAC03 affinity to a solid hydrophobic surface such as polystyrene. During the test, an aliquot of 1 mL of 1 × 10^8^ CFU/mL rehydrated bacterial suspension was placed in a 12-well polystyrene plate and incubated at 37 °C under anaerobic conditions. After one hour, the cultures were carefully removed, and the wells were washed three times with 1X PBS to remove the non-attached cells. The cells attached to the polystyrene were detached from the surface by cooling at 10 °C for 60 min, subsequently resuspended in 1X PBS, and plate-counted on MRS agar in triplicate. *Lp. plantarum* HAC03 fresh cells were used as controls.

### 2.12. Statistical Analysis

To determine the statistical significance of differences between the treatments, the results of three independent experiments were analyzed. Statistical analysis is described in each figure legend and was determined using GraphPad Prism 6 software (San Diego, CA, USA). One-way ANOVA was used for comparing more than 3 groups. Differences were adjudicated using the post hoc analysis recommended by PRISM. Statistical outliers were determined via the ROUT test (*p <* 0.05) and removed. *p <* 0.05 was considered to be statistically significant.

## 3. Results

### 3.1. Influence of Single Food Additives on Lp. plantarum HAC03 Viability after GIT Stress, Mucin Adhesion and Zeta Potential

The viability results showed that the carbohydrates generally exhibited the highest protection of *Lp. plantarum* HAC03 freeze-dried cells under conditions resembling passage of the upper GIT (*p <* 0.001) (Table 2). In cases of cells rehydrated in the presence of 1% glucose, viability levels were similar to those of fresh cultured bacteria (23.50% and 24.51%, respectively). The high viability levels obtained with glucose followed those found when either sorbitol (16.69%), sucrose (14.52%), mannose (13.48%), fructose (13.76%), or maltose (9.59%) were used. Proteins and nitrogenated molecules such as pepsin and soy peptone were also able to support the viability of *Lp. plantarum* HAC03 freeze-dried cells, but to a minor degree, with 7.30% and 14.43%, respectively (*p <* 0.01), next to albumin (6.46%) and yeast extract (17.27) (*p <* 0.05). Arabinose (0.66%), alginic acid (0.003%), arginine (0.04%), ornithine (0.84%), glutamic acid (0.10%), sodium L-tartrate (0.003%), malic acid (0.003%), pyruvic acid (0.003%), and ascorbic acid (0.10%) significantly affected the viability of the freeze-dried cells compared to rehydration only with distilled water (2.10%) (Table 2). The additives exerting low protection on bacterial viability were not further analyzed for mucin adhesion.

Regarding adhesion to mucin, again the carbohydrates showed the most beneficial influence on the freeze-dried bacteria. Percentages of adhesion obtained for the rehydration of freeze-dried bacteria were superior with mannose (18.80%), maltose (9.74%), glucose (6.50%), and fructose (5.79%), compared to values obtained for freshly cultivated bacteria (4.38%), and were significantly higher compared to rehydration of the freeze-dried bacteria with only distilled water (1.55%), *p <* 0.001 (Table 2). Yeast extract and tyrosine were also able to support adhesion of the dried bacteria to mucin, with respective levels of 3.23% and 6.89% (*p <* 0.001), followed by histidine (2.86% (*p <* 0.01)) and pepsin (2.45% (*p <* 0.05)). Compared to phenylalanine (0.14%), *p <* 0.001, taurine (0.48%) and thiamine (0.63%), (*p <* 0.01), and arabinose (0.84%), rhamnose (0.76%), trehalose (0.69%), albumin (0.83%), threonine (0.91%), sodium L-tartrate (0.72%), sodium bicarbonate (0.83%), betaine (0.67%) and riboflavin (0.81%) (*p <* 0.05) significantly affected adhesion of the freeze-dried bacteria to mucin.

A zeta potential of 21.03 mV was measured for freeze-dried *Lp. plantarum* HAC03, compared to −17.97 mV for freshly cultured cells. Contrary to the influence on viability and mucin adhesion, amino acids and proteinaceous molecules supported better re-establishing or maintenance of the zeta potential of the freeze-dried bacteria closer to the negative level of the fresh cells. Pepsin most strongly decreased the zeta potential of the freeze-dried bacteria to −11.57 mV, followed by yeast extract (−8.58 mV), sodium phosphate (−5.53 mV), sodium L-tartrate (−4.26 mV), soy peptone (−2.66 mV), histidine and riboflavin (−0.20 mV) (*p <* 0.001). Lysin (0.00 mV), ornithine (0.09 mV), glutamic acid (0.26 mV), arginine (0.74 mV), starch (2.27 mV), pyruvic acid (2.54 mV), sodium bicarbonate (2.69 mV), thiamine (3.47 mV), tyrosine (4.70 mV), serine (8.08 mV), betaine (10.05 mV), *p <* 0.001, phenylalanine (3.44 mV), threonine (10.68 mV), proline (14.60 mV), taurine (14.73 mV) (*p <* 0.01), and aspartic acid (17.10 mV) (*p <* 0.05), also reduced the zeta potential of the freeze-dried bacteria to a positive level or close to zero. On the other hand, arabinose and mannose significantly increased the zeta potential to 24.73 mV and 24.90 mV (*p <* 0.05), respectively, as compared to rehydration of the freeze-dried cells with distilled water (Table 2).

### 3.2. Effect of the Formulation Mixtures on Lp. plantarum HAC03 Viability after SSDP

Based on viability, the mucin adhesion test, and the zeta potential results, eight food grade additives were selected and combined in different possible formulations to find optimal synergistic effects between the excipients and the bacteria. Sucrose and sorbitol were selected for supporting high bacterial viability, 14.52% and 16.69%, respectively. Glucose, besides supporting bacterial viability, 23.50%, enhanced mucin adhesion to 6.50%. Soy peptone sustained bacteria viability, 14.43%, and reduced the zeta potential (−2.66 mV). Sodium bicarbonate and riboflavin reduced the zeta potential to 2.69 mV and −0.20 mV, respectively, while tryptophan which increased the zeta potential to 26.63 mV was selected for comparison.

Ten different ingredient formulations were tested, of which the five best (A to E) are shown in Table 1. Three mixtures showed elevated support of bacterial survival, i.e., A (31.06%), B (36.35%) and C (31.06%), achieving a similar or higher performance compared to that of the fresh cells (27.24%). Formulation B (subsequently termed “B-active”) increased bacterial survivability to 36.35%, which was almost 10% higher than the fresh cultures. Against expectations, formulations E and D containing sodium bicarbonate showed lower protection effects. Formulation E seemed to protect the bacteria from the acid stress typical of the stomach; however, with 4.93% survival, it was not effective in maintaining the viability of the freeze-dried bacteria after bile salt stress. With a 0.28% survival rate, formulation D was the least effective in sustaining viability of the freeze-dried cells under acid and bile stress conditions. In summary, the main contribution of the B-active formulation in the survival capacity of *Lp. plantarum* HAC03 was in the protection against low pH conditions typical of the stomach, although without directly neutralizing the gastric acids, but possibly by a stronger buffering effect. In addition, the B-active formulation was assayed to adjust the ingredients level that better supported bacterial viability. The results (data not shown) indicated that the amount of each specific ingredient may be critical in the development of a new probiotic product.

### 3.3. Determination of Lp. plantarum HAC03 Viability by Flow Cytometry and Influence of the B-Active Formulation

**Figure 1:**
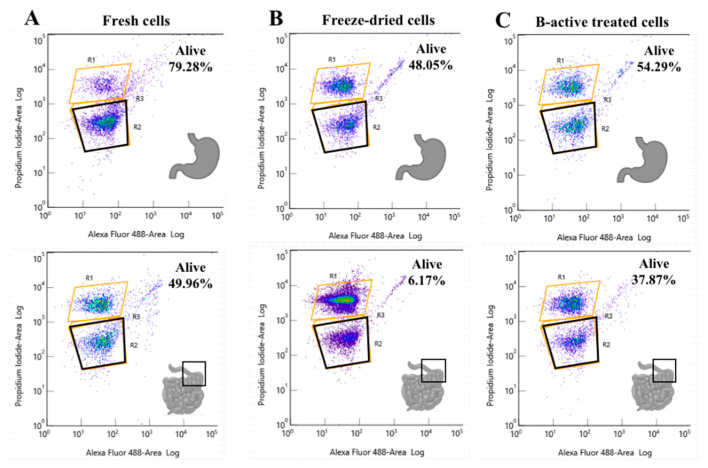
Real time detection of viability of *Lp. plantarum* HAC03 determined by flow cytometry after simulated stomach–duodenum passage. (**A**) *Lp. plantarum* HAC03 fresh cells cultured overnight in MRS broth at 37 °C. (**B**) Freeze-dried *Lp. plantarum* HAC03 cells rehydrated with 1 mL distilled water (DW) for 1 min at 25 °C. (**C**) Freeze-dried *Lp. plantarum* HAC03 cells mixed with B-active and rehydrated with 1 mL DW for 1 min at 25 °C. All live cells are gathered in the bottom black box, damaged cells in the lower orange box, and dead cells in the top orange box. The upper graphs represent HAC03 viability after the stomach passage, and the bottom graphs represent the viability after the duodenum passage. Viability tests were performed by a propidium iodine exclusion method.

In the previous experiments, *Lp. plantarum* HAC03 viability was determined by its ability to form colonies on MRS agar (plate counting). However, this test may be inadequate for slow-growing and/or viable but non-culturable cells. On the other hand, flow cytometry provides a tool for detecting total (live, dead, and damaged) bacterial cell numbers in real-time, and using propidium iodide dye provided a significant distinction between live and dead cell populations (Figure 1).

The results showed that after the GIT simulation, the viable numbers of *Lp. plantarum* HAC03 determined by plate counting corresponded to the data obtained by flow cytometry. The viability of freeze-dried cells rehydrated with DW was 6.17% determined by flow cytometry and 4.92% by plate counting (Figure 1 and Table 1). The flow cytometry results showed a viability of fresh cells and freeze-dried bacteria reactivated with B-active solution of 49.95% and 37.87%, respectively, compared to the 27.34% and 36.36% determined by plate counting (Figure 1 and Table 1). The differences in results between the plate counting and flow cytometry could be related to method sensitivity and to determining the counts with the flow cytometer in real time.

### 3.4. Impact of B-Active Formulation on Lp. plantarum HAC03 Zeta Potential and Hydrophobicity

**Figure 2:**
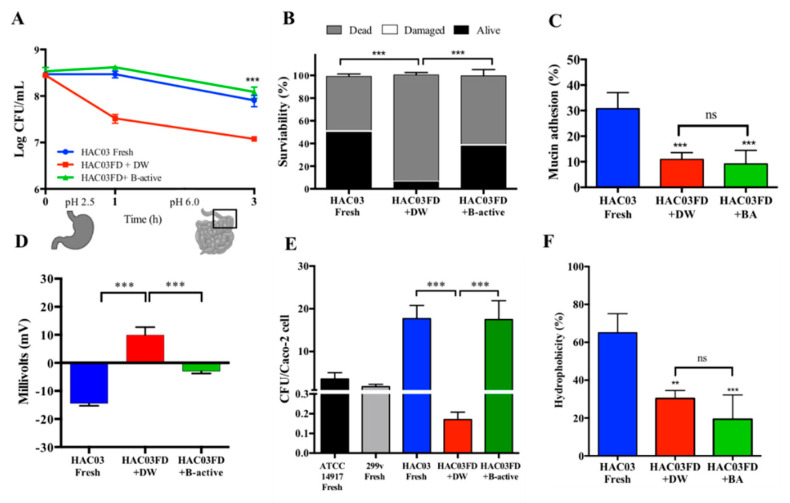
Comparison of freeze-dried *Lp. plantarum* HAC03 probiotic properties under different rehydration conditions. (**A**) Viability of *Lp. plantarum* HAC03 freeze-dried cells rehydrated in the presence of B-active (green line), without B-active (red line), and overnight cultured cells (blue line) after simulation of the GIT passage by plate counting. (**B**) Real-time *Lp. plantarum* HAC03 viability determined by flow cytometry and the exclusion method with propidium iodide. (**C**) *Lp. plantarum* HAC03 mucin adhesion, (**D**) zeta potential, (**E**) adhesion to Caco-2 cell line, and (**F**) hydrophobicity after rehydration with B-active formulation. *Lp. plantarum* ATCC14917 and *Lp. plantarum* 299v fresh overnight cultured cells were used as a reference and probiotic control, respectively. The experiments were carried out after rehydration and ten-fold dilution of the samples. Statistical differences were compared to HAC03 freeze-dried cells rehydrated with distilled water by one-way ANOVA and Dunnett’s multiple comparisons test, where ** *p <* 0.01, and *** *p <* 0.005.

Freeze-dried (and rehydrated in DW) and fresh cultures of *L. plantarum* HAC03 exhibited opposite (either negative or positive) surface charges or zeta potential values, respectively (Table 2). In order to find a possible connection between the decrease in mucin adhesion of the freeze-dried cells and the impact of the B-active formulation, the surface charge of *Lp. plantarum* HAC03 was measured. The results confirmed that B-active supported the re-establishment of the negative charge of the bacterial cells. These charges ranged from 9.87 mV for cultures rehydrated only with distilled water to −2.97 mV for cultures rehydrated with B-active formulation; this more closely resembled the value of −14.45 mV determined for fresh cultured bacteria (Figure 2D).

Hydrophobicity has also been linked to adhesive properties in bacteria; therefore, it was essential to determine if *Lp. plantarum* HAC03 hydrophobicity was also affected and modified after freeze-drying. The results indicate that *Lp. plantarum* HAC03 hydrophobicity was significantly (*p <* 0.01) decreased after freeze-drying, having percentages of hydrophobicity of 65.02% for the fresh cultured *Lp. plantarum* HAC03 cells compared to 30.29% for the freeze-dried cells that were rehydrated only with distilled water (Figure 2F). On the other hand, *Lp. plantarum* HACO3 freeze-dried cells showed the lowest hydrophobicity percentage (19.39%) in the B-active formulation, however this was not significantly different from the freeze-dried cells rehydrated with distilled water.

### 3.5. Influence of B-Active Formulation on the Adhesion of Lp. plantarum HAC03 to Intestinal Epithelial Cells and Mucin

Rehydration in the presence of B-active formulation significantly (*p <* 0.05) improved *Lp. plantarum* HAC03 adhesion (17.00 lactobacilli/cell) to Caco-2/TC-7 cells after simulated gastrointestinal passage compared to freeze-dried cells rehydrated with distilled water (equal to 0.17 lactobacilli/cell) and fresh cultures of the two control strains, *Lp. plantarum* 299v (1.79 lactobacilli/cell) and *Lp. plantarum* ATCC 14917 (3.6 lactobacilli/cell), respectively. Freeze-dried cultures of *Lp. plantarum* HAC03 rehydrated in the presence of B-active formulation exhibited a non-significantly different adhesion ability compared to fresh cultures of *Lp. plantarum* HAC03 (17.71 lactobacilli/cell).

On the other hand, *Lp. plantarum* HAC03 ability of mucin adhesion was lost in freeze-dried cells and was not recovered, even after rehydration with B-active formulation, maintaining percentages of adhesion similar to those observed in freeze-dried cells rehydrated with distilled water (Figure 2C).

### 3.6. Effect of B-Active Formulation on the Immunomodulatory Potential of Lp. plantarum HAC03

**Figure 3:**
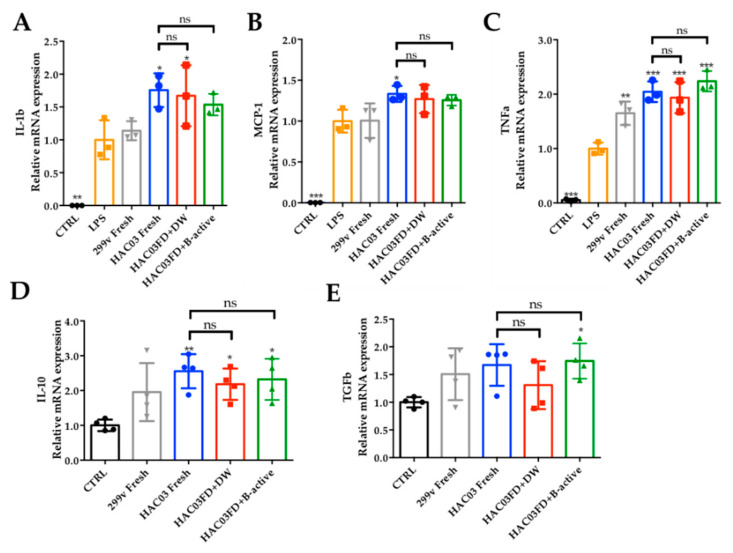
*Lp. plantarum* HAC03 immunomodulation in a murine macrophage cell line. Pro-inflammatory cytokines (**A**) interleukin 1 beta, (**B**) monocyte chemoattractant protein-1 and (**C**) tumor necrosis factor alpha and anti-inflammatory cytokines, (**D**) interleukin 10, and (**E**) transforming growth factor beta. Relative gene transcription levels (compared to the control) in a mouse macrophage cell line after 16 h stimulation with fresh and freeze-dried HAC03 cultures rehydrated with and without B-active. Fresh cultures were grown overnight on MRS broth at 37 °C, and freeze-dried bacteria were rehydrated with distilled water for 1 min at 25 °C before the test. Macrophages cultivated in DMEM media were used as negative controls (CTRLs), and cells treated with LPS (1 µg/mL) were used as a positive control. *Lp*. *plantarum* 299v was used as a probiotic control strain. Data are expressed as mean ± SD, *n* = 3. * *p* < 0.05, ** *p* < 0.01 and *** *p <* 0.005. compared to LPS and CTRL; one-way ANOVA, Dunnett’s multiple comparison test.

Cell adhesion is considered a key factor for interaction with the host’s GIT lumen, and thereby also in immunomodulation; this ability of *Lp. plantarum* HAC03 was reduced by freeze-drying. The impact of the freeze-drying process on the immunomodulatory features of the studied strains was investigated by determining the relative transcription of genes related to pro-inflammatory and anti-inflammatory cytokines in a mouse macrophage cell line. Macrophage cells non-treated and treated with lipopolysaccharide (LPS) were used as negative and positive controls, respectively, while fresh cells of *Lp. plantarum* 299 v were used as positive probiotic controls, and fresh and freeze-dried *Lp. plantarum* HAC03 cells rehydrated with and without B-active formulation were used as the test strain. The results suggest that there was no significant difference (*p* > 0.05) in the ability of fresh and freeze-dried *Lp. plantarum* HAC03 cells, with and without B-active formulation, to induce the production of pro-inflammatory and anti-inflammatory cytokines in the macrophage cell line. However, compared to the fresh culture, a slight reduction in the relative transcription of the anti-inflammatory cytokine interleukin 10 (IL-10) and the transforming growth factor beta (TGFb) (Figure 3D,E) were detected for HAC03 freeze-dried cells after rehydration with DW; this was notably improved for TGFb in the B-active formulation. On the other hand, *Lp. plantarum* 299v induced the lowest transcription of interleukin 1 beta (IL-1b), monocyte chemoattractant protein-1 (MCP-1), and tumor necrosis factor alpha (TNFa), pro-inflammatory cytokines, when compared to the transcription levels induced by *Lp. plantarum* HAC03 (Figure 3A–C).

### 3.7. Influence of B-Active Formulation on the Viability of Different Potential Probiotic Strains

**Figure 4:**
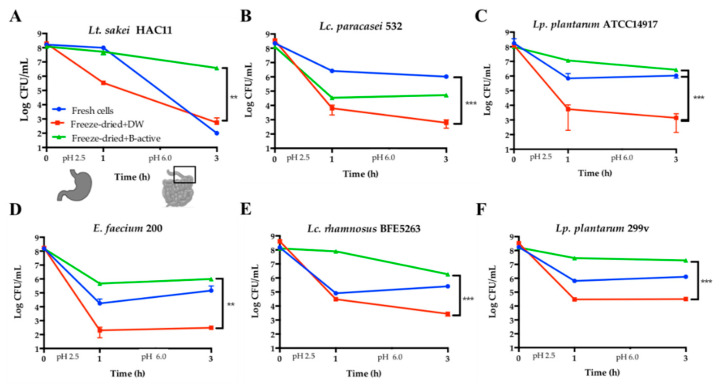
B-active impact on the survivability of different freeze-dried putative probiotic strains, compared to fresh cultures and cells rehydrated with distilled water. (**A**) *Latilactobacillus* (*Lt.*) *sakei* HAC11; (**B**) *Lc. paracasei* 532; (**C**) *Lp. plantarum* ATCC14917; (**D**) *E. faecium* 200; (**E**) *Lc. rhamnosus* BFE5263; and (**F**) *Lp. plantarum* 299v, viability of fresh overnight cells (blue line), freeze-dried cells rehydrated only with distilled water (red line), and freeze-dried cells rehydrated with B-active (green line) after simulated gastro-intestinal passage (SSDP). Data are expressed as the mean ± SD, *n* = 3. ** *p <* 0.01 and *** *p <* 0.005; one-way ANOVA, Dunnett’s multiple comparison test.

The efficacy of the B-active formulation for increasing bacterial survivability after the complete SSDP was also tested for other putative probiotic strains such as *Latilactobacillus (Lt.) sakei* HAC11, *Lc. paracasei* 532, *Lp. plantarum* ATCC 14917, *Enterococcus faecium* 200, *Lc. rhamnosus* BFE5263 and *Lp. plantarum* 299 v. The results showed that B-active formula also effectively protects all these strains. In most cases, except for *Lc. paracasei* 532, freeze-dried cells rehydrated with B-active formulation reached higher viabilities compared to their corresponding fresh cultures (Figure 4). In addition, dried cells of *Lt. sakei* HAC11 and *Lp. plantarum* ATCC 14917 exhibited up to 4-log higher viability after SSDP as a result of rehydration with B-active formulation, as compared to rehydration only with distilled water. Furthermore, 3-log higher levels of *E. faecium* 200, *Lc. rhamnosus* BFE5263 and *Lp. plantarum* 299 v and only up to 2-log higher numbers for *Lc. paracasei* 532 were detected (Figure 4).

## 4. Discussion

The current scientific consensus definition states that probiotics should be alive in order to exert their beneficial effects in the human GIT [48]. Viability of bacterial cells will be reduced when subjected to sub-lethal stresses (e.g., variations in pH, low temperature, ice crystal formation) during the freeze-drying process. In addition, surviving and partially impaired cells will be more sensitive to stress factors such as increased osmotic pressure and physiological conditions in the human GIT after ingestion [1,17,48]. Commercial probiotics in powder form are usually not rehydrated before consumption. Lyophilization may cause sublethal injury of a strain, and its vitality in powdered form may further be reduced by direct intake and exposure to stress factors in the upper intestinal tract. Additionally, rapid transit from the dormant state to reactivation under ecological stress conditions typical of the upper GIT may further limit cellular repair. On the other hand, in the case of rehydration, reconstitution is usually with excessive water, more than that removed during the dehydration process, thereby resulting in stress effects due to reduced osmotic pressure. Rehydration is therefore a highly critical step in the revitalization of a lyophilized culture [21]. The appropriate selection of components that favor optimal cellular rehydration may decisively influence its eventual functionality [49]. According to declarations on the label of some probiotics commercialized in Korea and the Philippines, the main ingredients used as excipients comprise sugars, amino acids, vitamins, minerals, salts, starch, FOS, and inulin. (Table S2). Although some of these products/ingredients can be considered as prebiotics and/or growth factors, their constituents might not be suitable for supporting the viability and/or reactivation of the freeze-dried microbial cells, and they can have different roles in formulation of the pharmaceutical product.

In an attempt to formulate a product to maximize bacterial viability and/or support probiotic function, the compatibility of 40 different food additives approved by the U.S. FDA was evaluated for supporting viability, mucin adhesion, and zeta potential after the rehydration of freeze-dried *Lp. plantarum* HAC03. The viability results indicated that carbohydrates, proteins, and nitrogenated molecules generally provided better protection for freeze-dried bacteria after rehydration and subjection to the simulated harsh environment of the upper GIT (*p <* 0.001). Corcoran et al. [50] explained that glucose can be involved in the process that provides ATP to F0F1-ATPase via glycolysis, thereby allowing the exclusion of protons from the cells during exposure to simulated gastric juice at pH 2.0. The permeability of the different additives could also be related either to their level of protection on the cells or the mechanisms involved. Small molecules such as glycerol are able to penetrate both the cell wall and the cytoplasmatic membrane. However, oligosaccharides, amino acids, and low molecular weight biopolymers are able only to penetrate the cell wall but not the cytoplasmic membrane. On the other hand, polymers with high molecular weight, such as proteins and polysaccharides, can neither penetrate the cell wall nor have direct interaction with the cell wall or membrane. For instance, proteinaceous components, e.g., from skim milk, can stabilize the cell membrane constituents and provide a protective coating for the cells during freeze drying [51], while amino acids react with carboxyl groups of the bacterial proteins and thereby stabilize protein structure [4].

The different degrees of protection to the freeze-dried cells by the rehydration components could also be related to differences in the control of water flux into the cells by the rehydrating media [18].

The effects of ingredients for providing stronger protection were especially pronounced during pH stress. Thus, the pH buffering effect of some ingredients may be one possible mechanism involved in the maintenance of cell homeostasis during rehydration. The low pH of the gastric juices mainly disrupts the membrane transport by affecting the proton motive force, denaturing acid-sensitive enzymes, and triggering the inhibition of specific physiological functions [1]. A previous report [1] stated that pH stress can be the strongest determinant of reduced viability of bacteria in the rehydration process. On the other hand, under small intestinal conditions, the microorganism’s vulnerability derives from the bile salts which are associated with disruption of the cell membranes and the lowering of the intracellular pH by the release of protons [52].

The carbohydrates had the most favorable effect on adhesion of the freeze-dried bacterial cells to mucin. Du Toit et al. [53] also reported that freeze-drying significantly decreased the adhesion of *Lc. rhamnosus* GG, *Bifidobacterium lactis* Bb-12 and *Bifidobacterium animalis* IF20/1, but not of *Lc. casei* Shirota to human colonic mucus, and also reduced their ability to exclude or displace pathogens. This highlights the fact that such effects are strain-dependent, and some strains appear to be more sensitive to the lyophilization process in relation to their future application. Mucin-binding proteins (Mub) are surface proteins linked to peptidoglycan in the bacterial cell wall and can interact with mucin sugar residues. Although mucins are found in several bacteria species, the Mub domains are almost exclusive for LAB of gastrointestinal origin. Pili and other surface structures such as fibronectin-binding proteins and surface layer proteins (S-layer) have also been involved in bacterial adhesion to the intestinal mucosa [54], and we could assume that these surface structures could be damaged during the freeze-drying process. Thus, carbohydrates used as food additives could interact with the mucin sugar residues and thereby enhance bacterial adhesion to mucin.

Finally, amino acidic and proteinaceous molecules were the ones that better re-established the zeta potential of freeze-dried bacteria closer to the negative values observed for the fresh cells. Zeta potential is determined by the nature of the groups displayed on the surface; under physiological (active) conditions, bacteria are usually negatively charged due to the large amount of phosphate and carboxyl groups present on the cell surface [33]. Soni et al. (2007) [55] highlighted the importance of the physiological state for the bacterial surface charge, because starved *E. coli* and *Salmonella* Newport cells exhibited a lower zeta potential compared to cells grown under rich conditions. In 2017, Ng and Ting [56] reported that adsorption of ions or polyelectrolytes onto the cell surface resulted in modification of the bacterial surface charge and polarity, as was reflected by the zeta potential values. Additives with either a proteinaceous or amino acidic nature might have a higher impact on bacterial charge because of the protonation of amino, carboxyl, and phosphate groups at low pH [33], while the sugars seem to be less reactive.

Among the tested formulations, the one referred to as B-active, composed of sucrose, sorbitol, and soy peptone with some traces of malic acid, showed the highest efficacy on revitalization of *Lp. plantarum* HAC03 freeze-dried cells during the rehydration process. Costa et al. [57] previously reported a significant increase in freeze-dried bacterial recovery after rehydration in a complex medium containing skim milk, peptone, tryptone, meat extract and sucrose, compared to buffer phosphate and sodium glutamate solutions and water. High viability in streptococci has also been reported for a rehydration medium containing sugars such as sucrose and dextrose and skim milk proteins; however, by comparison, a minimal number of viable cells was recovered when DW was used as rehydrating medium [58]. For all experiments in this study, we have rehydrated *Lp. plantarum* HAC03 in DW as a control. Elevated cell injury during rehydration with DW in our study could be explained by the osmotic imbalance caused by the exposure of cells to hypotonic solutions and the subsequent exposure to low pH. In addition, the freezing step during lyophilization renders the lipids on the cell membrane more susceptible to damage, this being important to determine the ability of microorganisms to tolerate stressing environmental conditions, such as acidity and bile salts, and to evaluate both the metabolic capacity and probiotic functionality after freeze-drying [59]. In addition, an increase in bacterial sensitivity to agents such as salts, acid, enzymes, and antibiotics has been associated and used as a measurement of membrane damage [60].

Sugars are an easily available source of energy; glucose and some other monosaccharides, particularly, can be utilized right after rehydration and thus support bacterial viability under stressful conditions. Sugars such as sorbitol, maltose, and mannitol have been effective in the protection of bacteria against oxidation over long periods of storage. The mechanism behind the antioxidant protection of sugars could be related to their ability to react with hydrogen peroxide and restrict oxygen diffusion [61]. Additional mechanisms involved in the sorbitol protection of dried cells could be related to the formation of sorbitol–protein complexes that stabilize protein structures, thus preserving their functionality [62]. In addition, the use of sugars not only during freeze-drying but also during storage have resulted in higher bacteria survival at room temperatures [61]. The viability of *Lactobacillus bulgaricus*, *Lp. plantarum*, *Lc. rhamnosus*, *E. faecalis* and *E. durans* was strongly supported by sorbitol during storage, despite the lack of impact on the viability during freeze-drying [63].

The capacity of probiotics to adhere to cell-lines and persist despite the peristaltic movements of the colon may contribute to colonization and enhance the interaction with immune cells and the gut microbiota [64]. Adhesion is a complex process that involves interactions between the bacteria and the union surface. LAB adhesion to intestinal epithelial cells has been associated with surface layer proteins and structures as well as cell surface charge and bacterial cell hydrophobicity [65]. Zeta potential is a parameter that estimates bacterial surface charge and is defined as the electric charge at the shear plane [56]. De Wouters et al. [34] suggested the use of zeta potential together with interfacial viscoelasticity and interfacial tension to characterize the surface properties of intestinal bacteria and predict their adhesion potential in the GIT. In the same study [34], conducted with *Lc. rhamnosus* GG and *Lc. rhamnosus* DSM 20021T, the influence of surface proteins, cell hydrophobicity, and electric charge on adhesive properties was confirmed. Our results indicate that a positive change in bacterial zeta potential can be partially explained by a reduction in the adhesive properties of the freeze-dried cells. The zeta potential of the *Lp. plantarum* HAC03 cells recovered in the presence of the B-active formulation may be related to the interaction of the bacterial cell surface groups with the sugars, proteins and ingredients present in the formulation. This may eventually have contributed to the increase in bacteria cell adhesion. Modification of the zeta potential has been used before to inhibit the initial adhesion of *Streptococcus mutans* as a strategy to prevent dental caries [66].

During the drying process, the number of biological oxidation reactions increases due to the formation of free radicals. Thereby, the hydrophobicity of fatty acids is reduced and, as a consequence of the introduction of hydrophilic groups, the hydrophobic interactions with proteins of the membrane will deteriorate [67]. The adhesiveness of bacteria to hydrocarbons has been used to express cell surface hydrophobicity. Hydrophobicity is considered a key factor in the adhesion of bacteria to intestinal epithelial cells and is responsible for the strongest long-range non-covalent interactions [68]. Surface proteins such as cell wall-anchored proteinases have been shown to be responsible for increased hydrophobicity and LAB adhesion [54]. However, our results show that the B-active mixture did not influence the hydrophobicity of the cells despite the inclusion of ingredients with antioxidant properties.

Preservation processers such as spray-drying have reduced the adhesion of *Lc. rhamnosus* GG six-fold, and scanning electron micrographs revealed that the drying process sheared off bacterial pili, a key surface factor for adherence to intestinal cells and mucus [13]. Lebeer et al. [69] demonstrated the importance of *Lc. rhamnosus* GG SpaCBA pili as a mediator of adhesion to Caco-2 cells in vitro. Damage of surface molecules essential for bacterial adhesion or indispensable for probiotic interaction with the host might affect their efficacy, even when the bacterial numbers are maintained. The adhesiveness of *Lentilactobacillus (Lt.) kefiri* 8348 to intestinal cells was also reduced after spray-drying, even though no injuries were detected on the cell membrane; however, similar effects have not been observed for *Lp. plantarum* 83114 or *Lt. kefiri* 8321 [60]. Although *Lt. kefiri* 8321 maintained the ability to adhere to intestinal cells, its capacity of protection against *Salmonella* invasion was reduced by spray-drying. Structural damage of the surface proteins during the drying process was suggested as an explanation, because S-layer proteins have been linked with *Salmonella* antagonism in a previous study [70]. The ingredients of the B-active formulation seem to favor the adhesion of freeze-dried bacteria to intestinal epithelial cells by linking the microorganisms to the epithelial cells, although such a mechanism still needs to be elucidated. Thus, B-active may also potentially support the maintenance of putative probiotic properties such as adhesion to intestinal cells.

Previous reports have demonstrated that probiotics may induce immune-modulatory responses in the intestine by the production of cytokines during interaction with host immune cells [71]. Bacterial structures involved in adhesion, such as S-layer proteins, can also interact with host receptors in the intestine and produce immunological response [72]. A study on macrophages revealed the importance of *Lc. rhamnosus* GG SpaCBA pili in the induction of IL-10 and the reduction in IL-6 gene transcription [73]. Our results indicate that the immunomodulatory properties of *Lp. plantarum* HAC03 were not significantly modified after the freeze-drying process, or after its interaction with the B-active formulation. Iaconelli et al. [12] evaluated the impact of air-drying, freeze-drying, and spray-drying on *B. bifidum*, *Lp. plantarum* and *Lc. zeae* immuno-stimulatory properties in human peripheral blood mononuclear cells and observed non-significant differences in the anti-inflammatory response measured by IL-10 production of the mononuclear cells treated with fresh or rehydrated bacteria; however, there was a reduction in the pro-inflammatory response (IL-12 production) of *Lc. zeae* and *B. bifidum*.

As indicated by the results, B-active formulation also supported the viability of diverse species and strains of freeze-dried putative probiotics to different levels. Such differences could be related to genetic, cell-wall, and membrane compositional differences, and/or to underlying mechanisms that are not completely understood but also might influence strain-specific tolerance to several stress conditions [6]. Bacteria usually respond to different stress levels related to the environment from which they were isolated and to the time and prior stress conditions to which they have been exposed [74]. Important factors involved include population density-related bacterial quorum sensing and responses to environmental stress such as the two-component signal transduction system which senses conditions in the environment and transfers a signal that elicits response by affecting gene transcription [75,76,77].

## 5. Conclusions

Deterioration of a probiotic strain during in vivo gastro-intestinal passage is determined by a range of stress factor. It starts in the mouth and continues throughout the GIT, resulting in a reduction in its functional potential. The careful selection of appropriate excipients when formulating new probiotic supplements represents a vital step towards the sustaining of vitality and physiological activity of sub-lethally damaged cells in freeze-dried matrices. Major requirements for such formulations are the protection of cells against osmotic shock during rehydration, and the maintenance or enhancement of probiotic viability and functionality during GIT passage without compromising a strain’s key probiotic characteristics. Our data suggest that the B-active formulation supports the maintenance of the integrity of the tested probiotic candidate strains under simulated stress conditions of the GIT. Additional studies may be necessary to (a) further optimize the selected B-active formulation, and (b) elucidate underlying mechanisms involved in the protection of homeostasis of freeze-dried bacteria during and after rehydration.

## 6. Patents

Patent number 10-2020-0044762, Korea.

## Figures and Tables

**Table 1:** Survival of freeze-dried *Lp. plantarum* HAC03 after passing the simulated environment of the GIT as a function of rehydration with five formulations containing different proportions of selected cell-protecting food additives.

		Log CFU/mL	Log CFU/mL	Survival (%)	Log CFU/mL	Survival (%)
Fresh cells *Lp. plantarum* HAC03		8.47 ± 0.01	8.47 ± 0.07	99.83	7.90 ± 0.12	27.24 ***
Freeze-dried cells HAC03+DW		8.48 ± 0.04	7.57 ± 0.09	12.24	7.17 ± 0.11	4.92
A	Sucrose	8.46 ± 0.12	8.46 ± 0.18	104.77	7.94 ± 0.18	31.06 ***
	Malic acid					
	Sorbitol					
	Soy peptone					
	Riboflavin					
B	Sucrose	8.51 ± 0.07	8.44 ± 0.06	84.77	8.07 ± 0.05	36.35 ***
	Malic acid					
	Sorbitol					
	Soy peptone					
C	Sucrose	8.42 ± 0.03	8.28 ± 0.06	71.50	7.91 ± 0.01	31.06 ***
	Sorbitol					
	Soy peptone					
D	Soy peptone	8.66 ± 0.05	8.27 ± 0.02	40.82	6.10 ± 0.04	0.28
	Tryptophan					
	Sorbitol					
	Sodium bicarbonate					
E	Soy peptone	8.51 ± 0.23	8.57 ± 0.06	120.89	7.17 ± 0.01	4.93
	Tryptophan					
	Glucose					
	Sodium bicarbonate					

*** *p <* 0.001.

**Table 2:** Effect of rehydration with different food additives on the viability, zeta potential and mucin adhesion of freeze-dried *Lp. plantarum* HAC03 after subjection to simulated GIT conditions.

Treatment		Initial Counts	After 1 h Acid Stress		After 2 h Bile Stress		Mucin Adhesion (%)	Zeta Potential (mV)
		Log CFU/mL	Log CFU/mL	Survival (%)	Log CFU/mL	Survival (%)		
Fresh cells of *Lp. plantarum* HACO3		8.38 ± 0.13	8.07 ± 0.05	50.30	7.77 ± 0.13	24.51 ***	4.38 ± 0.38 ***	−17.97 ± 1.12 ***
Freeze-dried cells of HAC03 + DW		7.99 ± 0.12	6.71 ± 0.17	5.40	6.27 ± 0.28	2.10	1.55 ± 0.36	21.03 ± 1.46
1	Arabinose	8.09 ± 0.04	6.55 ± 0.02	2.91	5.90 ± 0.13	0.66 **	0.84 ± 0.00 *	24.73 ± 1.20 *
2	Xylose	7.95 ± 0.03	6.74 ± 0.32	7.12	6.20 ± 0.37	2.15	1.57 ± 0.05	23.13 ± 1.24
3	Rhamnose	8.00 ± 0.01	6.62 ± 0.09	4.28	6.14 ± 0.12	1.44	0.76 ± 0.03 *	23.13 ± 2.11
4	Mannose	8.02 ± 0.01	7.80 ± 0.02	60.21	7.14 ± 0.07	13.48 ***	18.80 ± 0.68 ***	24.90 ± 1.64 *
5	Fructose	8.07 ± 0.12	7.66 ± 0.03	39.24	7.20 ± 0.25	13.76 ***	5.79 ± 0.21 ***	24.77 ± 2.33
6	Mannitol	8.02 ± 0.01	6.91 ± 0.18	8.02	6.47 ± 0.07	2.80	0.99 ± 0.02	22.70 ± 1.11
7	Sucrose	7.95 ± 0.03	7.63 ± 0.06	48.25	7.07 ± 0.28	14.52 ***	1.01 ± 0.07	21.60 ± 1.28
8	Sorbitol	8.00 ± 0.05	7.54 ± 0.09	34.75	7.22 ± 0.03	16.69 ***	1.45 ± 0.01	23.17 ± 1.12
9	Glucose	7.93 ± 0.04	7.60 ± 0.10	48.07	7.30 ± 0.02	23.50 ***	6.50 ± 0.37 ***	23.10 ± 1.67
10	Maltose	8.19 ± 0.16	7.72 ± 0.04	35.75	7.15 ± 0.16	9.39 ***	9.74 ± 0.14 ***	21.03 ± 1.16
11	Trehalose	7.94 ± 0.04	6.52 ± 0.24	3.95	6.13 ± 0.11	1.54	0.69 ± 0.04 *	22.87 ± 1.40
12	Alginic acid	8.00 ± 0.00	4.00 ± 0.00	0.01	3.49 ± 0.00	0.003 **	nd	−4.86 ± 1.24 ***
13	Starch	8.10 ± 0.10	6.47 ± 0.13	2.51	6.16 ± 0.04	1.18	0.26 ± 0.02 **	2.27 ± 0.89 ***
14	Gelatin	8.10 ± 0.01	6.89 ± 0.13	6.22	6.68 ± 0.37	4.48	2.01 ± 0.94	23.40 ± 3.46
15	Albumin	8.11 ± 0.18	6.93 ± 0.05	7.11	6.85 ± 0.17	6.46 *	0.83 ± 0.07 *	28.73 ± 2.20 **
16	Pepsin	8.04 ± 0.04	7.16 ± 0.09	13.29	6.89 ± 0.14	7.30 **	2.45 ± 0.14 *	−11.57 ± 1.14 ***
17	Soy Peptone	8.23 ± 0.04	7.53 ± 0.38	24.86	7.31 ± 0.33	14.43 **	1.42 ± 0.16	−2.66 ± 0.43 ***
18	Yeast extract	8.11 ± 0.06	7.36 ± 0.72	29.65	7.11 ± 0.75	17.27 *	3.23 ± 0.10 ***	−8.58 ± 0.39 ***
19	Arginine	7.44 ± 0.79	4.00 ± 0.00	0.07	3.61 ± 0.17	0.04 **	nd	0.74 ± 0.10 ***
20	Tryptophan	8.12 ± 0.02	6.42 ± 0.08	1.99	6.14 ± 0.04	1.05	1.69 ± 0.19	26.63 ± 0.25 **
21	Phenyl alanine	8.00 ± 0.02	6.53 ± 0.05	3.41	6.15 ± 0.13	1.44	0.14 ± 0.001 ***	3.44 ± 6.10 **
22	Ornithine	8.10 ± 0.02	6.47 ± 0.38	2.78	6.01 ± 0.16	0.84 *	1.44 ± 0.15	0.09 ± 0.15 ***
23	Glutamic acid	8.03 ± 0.03	6.87 ± 0.91	26.93	4.19 ± 1.09	0.10 **	nd	0.26 ± 0.20 ***
24	Proline	8.14 ± 0.07	6.91 ± 0.04	5.91	6.45 ± 0.22	2.24	1.32 ± 0.40	14.60 ± 1.21 **
25	Lysine	8.01 ± 0.24	6.05 ± 0.37	1.16	5.72 ± 0.41	0.54	0.97 ± 0.09	0.00 ± 0.16 ***
26	Serine	7.99 ± 0.20	6.58 ± 0.18	3.92	6.26 ± 0.13	1.87	1.85 ± 0.11	8.08 ± 0.96 ***
27	Threonine	7.87 ± 0.03	6.52 ± 0.13	4.62	6.15 ± 0.10	1.97	0.91 ± 0.10 *	10.68 ± 2.14 **
28	Aspartic Acid	7.84 ± 0.05	7.06 ± 0.69	29.95	5.80 ± 0.78	1.86	nd	17.10 ± 0.82 *
29	Tyrosine	7.68 ± 0.30	6.08 ± 0.57	3.12	5.84 ± 0.61	1.82	6.89 ± 0.16 ***	4.70 ± 1.35 ***
30	Histidine	7.70 ± 0.12	6.27 ± 0.01	3.86	5.87 ± 0.05	1.57	2.86 ± 0.60 **	−0.20 ± 0.67 ***
31	Sodium phosphate	7.87 ± 0.00	6.29 ± 0.04	2.66	6.01 ± 0.09	1.39	0.96 ± 0.04	−5.53 ± 0.34 ***
32	Sodium L-tartrate	8.03 ± 0.19	4.00 ± 0.00	0.01	3.49 ± 0.00	0.003 *	0.72 ± 0.05 *	−4.26 ± 1.19 ***
33	Sodium bicarbonate	8.08 ± 0.02	6.56 ± 0.02	3.06	6.04 ± 0.15	0.96	0.83 ± 0.04 *	2.69 ± 0.12 ***
34	Malic acid	8.00 ± 0.00	4.00 ± 0.00	0.01	3.49 ± 0.00	0.003 **	nd	21.57 ± 1.89
35	Pyruvic acid	8.00 ± 0.00	4.00 ± 0.00	0.01	3.49 ± 0.00	0.003 **	nd	2.54 ± 0.04 ***
36	Betaine	7.90 ± 0.04	6.57 ± 0.05	4.75	6.19 ± 0.06	1.95	0.67 ± 0.03 *	10.05 ± 1.34 ***
37	Taurine	7.87 ± 0.19	6.50 ± 0.17	4.35	6.25 ± 0.13	2.46	0.48 ± 0.01 **	14.73 ± 0.46 **
38	Riboflavin	7.96 ± 0.22	6.84 ± 0.16	7.70	6.22 ± 0.20	1.85	0.81 ± 0.14 *	−0.20 ± 1.43 ***
39	Thiamine	7.77 ± 0.32	6.41 ± 0.34	4.46	5.47 ± 0.33	0.50	0.63 ± 0.06 **	3.47 ± 0.40 ***
40	Ascorbic acid	6.49 ± 0.03	4.08 ± 0.11	0.40	3.49 ± 0.00	0.10 **	nd	21.07 ± 1.99

Fresh culture was grown overnight on MRS broth at 37 °C. Freeze-dried bacteria were mix with 0.001 M of each food additive and rehydrated with 1 mL distilled water for 1 min at 25 °C to a concentration of 1 M. Simulated GIT passage, zeta potential, and mucin adhesion tests were carried out after rehydration and after diluting of the samples to a final concentration of 0.1 M. Stomach conditions were simulated with PBS 1X at pH 2.5 (1 h acid stress). Duodenum pass was mimicked by a combination of duodenum juice containing NaCl, KCl, NaHCO_3_ and 10% ox-gall pH 6 (2 h bile stress). Zeta potential was measured on bacteria suspended in double-distilled water at pH 2.0. Mucin type II adhesion from porcine stomach was measured after 1 h incubation at 37 °C. Data are expressed as the mean ± SD, *n* = 3. * *p <* 0.05, ** *p <* 0.01 and *** *p <* 0.001 compared to freeze-dried cells rehydrated only in the presence of distilled water; one-way ANOVA, Dunnett’s multiple comparison test. nd: not determined.

## Supplementary Materials

The following are available online at https://www.mdpi.com/article/10.3390/microorganisms9051013/s1, Table S1: List of food additives according to the U.S. FDA and their major classified function; Table S2: Main components declared for selected commercial probiotics in Korea and the Philippines.
