# Supplementary material for: Rehydration before Application Improves Functional Properties of Lyophilized Lactiplantibacillus plantarum HAC03
Source: Microorganisms. 2021 May 8;9(5):1013. doi: 10.3390/microorganisms9051013 (PMC8150888; doi:10.3390/microorganisms9051013)
Supplement: Supplementary file 1 [file microorganisms-09-01013-s001.zip › microorganisms-1211650-supplementary.pdf]

microorganisms

MDPI

Supplementary Material

Table S1. List of food additives according to the US FDA and their major classified function.

Food additive	Formula	Molecular weight (g/mol)	Purity (%)	Weighted amount (g)	Rehydration concentration	Final test concentration	Brand	Lot	Major function class
Arabinose	C5H10O5	150,13	100,00	0,15	1M	0,1M	USB	70047,000	Flavor enhancer, flavoring agent or adjuvant
Xylose	C5H10O5	150,10	99,00	0,15	1M	0,1M	Sigma	053K00131	Flavoring agent or adjuvant, nutritive sweetener
Rhamnose	C6H12O5. H20	182,20	99,00	0,18	1M	0,1M	Sigma	058K0695	Flavoring agent or adjuvant
Mannose	C6H12O6	180,16	99,00	0,18	1M	0,1M	Sigma	BCBC4173V	Flavoring agent or adjuvant
Fructose	$C_6H_{12}O_6$	180,16	100,00	0,18	1M	0,1M	Sigma	125K01611	Nutritive sweetener
Mannitol	C6H14O6	182,17	100,00	0,18	1M	0,1M	Sigma	034K0061	Anticaking agent or free-flow agent, flavor enhancer, flavoring agent or adjuvant, formulation aid, lubricant or release agent, non-nutritive sweetener, solvent or vehicle
Sucrose	$C_{12}H_{22}O_{11}$	342,30	100,00	0,34	1M	0,1M	Daejung	S0784RE1	Nutritive sweetener
Sorbitol	$C_6H_{14}O_6$	182,17	97,00	0,19	1M	0,1M	Daejung	S0503QC1	Color or coloring adjunct, drying agent, flavoring agent or adjuvant, humectant, nutrient supplement, nutritive sweetener, pH control agent, solvent or vehicle, stabilizer or thickener, texturizer
Glucose	$C_6H_{12}O_6$	180,16	99,50	0,18	1M	0,1M	Sigma	SLBS2877V	Anticaking agent or free-flow agent, color or coloring adjunct, drying agent, flavor enhancer, flavoring agent or adjuvant, humectant, lubricant or release agent, nutritive sweetener, solvent or vehicle, stabilizer or thickener
Maltose	C12H22O11.H2O	360,31	100,00	0,36	1M	0,1M	USB	109469,000	Nutritive sweetener
Trehalose	C12H22O11.2H20	378,33	99,00	0,38	1M	0,1M	Sigma	SLBV3123	Flavoring agent or adjuvant
Alginic acid	$(C_6H_8O_6)_n$	10,00 – 600,00	100,00	0,30	1M	0,1M	Sigma	127H0923	Emulsifier, thickener, stabilizer
Starch	(C6H10O5)n	-	100,00	0,30	1M	0,1M	Daejung	S0371PG1	Anticaking agent or free-flow agent, drying agent, flavoring agent or adjuvant, formulation aid, humectant, non-nutritive sweetener, nutritive sweetener, solvent or vehicle, stabilizer or thickener, texturizer
Gelatin	-	-	-	0,02	2%	0,2%	Daejung	G0001PB1	Anticaking agent or free-flow agent, drying agent, flavoring agent or adjuvant, formulation aid, humectant, processing aid, solvent or vehicle, stabilizer or thickener, surface-finishing agent, texturizer
Albumin	-	-	-	0,02	2%	0,2%	Sigma	SLBQ9036V	Firming agent, formulation aid, nutrient supplement, processing aid, propellant, stabilizer or thickener, surface-active agent, surface-finishing agent, texturizer
Pepsin	-	-	-	0,02	2%	0,2%	Sigma	SLCC6249	Enzyme
Peptone Soy	-	-	-	0,02	2%	0,2%	Daejung	P2758OH1	Flavor enhancer, flavoring agent or adjuvant
Yeast extract	-	-	-	0,02	2%	0,2%	BD Bacto	8015615	Flavor enhancer, flavoring agent or adjuvant, malting or fermenting aid, nutrient supplement
L-Arginine	C6H14N4O2	174,20	98,00	0,18	1M	0,1M	Sigma	017K0664	Nutrient supplement
L-Tryptophan	C11H12N2O2	204,23	99,00	0,21	1M	0,1M	Kanto chemical co.	403N2187	Nutrient supplement
L-Phenylalanine	C9H11NO2	165,19	99,00	0,17	1M	0,1M	Junsei	2012L1433	Flavor enhancer, flavoring agent or adjuvant, nutrient supplement
L-Ornithine	C5H12N2O2.ClH	168,62	99,00	0,17	1M	0,1M	Sigma	109K1468	Flavoring agent or adjuvant
L- Glutamic acid	C5H8NNaO4.xH2O	169,11	99,00	0,17	1M	0,1M	Sigma	SLBF7449V	Flavoring agent or adjuvant, nutrient supplement
L-Proline	C5H9N02	115,10	100,00	0,12	1M	0,1M	Sigma	72H0774	Flavor enhancer, flavoring agent or adjuvant, nutrient supplement
L-Lysine	C6H14N2O2.HCl	182,65	98,00	0,19	1M	0,1M	Sigma	108K1321	Nutrient supplement
L-Serine	$C_3H_7NO_3$	105,09	99,00	0,11	1M	0,1M	Georgiachem	S454621R	Flavor enhancer, flavoring agent or adjuvant, nutrient supplement
L-Threonine	C4H9NO3	119,12	99,00	0,12	1M	0,1M	Georgiachem	T262958H	Flavor enhancer, flavoring agent or adjuvant, nutrient supplement
L-Aspartic Acid	C4H7NO4	133,10	99,00	0,13	1M	0,1M	Georgiachem	A977123A	Flavor enhancer, flavoring agent or adjuvant, nutrient supplement
L-Tyrosine	$C_9H_{11}NO_3$	181,19	99,00	0,18	1M	0,1M	Samchun chemicals	101917	Flavoring agent or adjuvant, nutrient supplement
L-Histidine	C6H9N3O2	209,64	99,00	0,21	1M	0,1M	Daejung	H2821RL1	Flavor enhancer, flavoring agent or adjuvant, nutrient supplement
Sodium Phosphate	$Na2HPO_4$	141,96	99,00	0,14	1M	0,1M	Sigma	075K2520	Anticaking agent or free-flow agent, antimicrobial agent, drying agent, emulsifier or emulsifier salt, flavor enhancer, flavoring agent or adjuvant, malting or fermenting aid, pH control agent, stabilizer or thickener, texturizer
Sodium L-tartrate	C4H4Na2O6.2H2O	230,08	99,00	0,23	1M	0,1M	Sigma	07425BC	Emulsifier or emulsifier salt, pH control agent, sequestrant
Sodium bicarbonate	NaHCO3	84,01	99,80	0,08	1M	0,1M	Yakuri	312132812	Emulsifier or emulsifier salt, flavor enhancer, flavoring agent or adjuvant, leavening agent, nutrient supplement, pH control agent, processing aid, solvent or vehicle
Malic acid	C4H6O5	134	98,00	0,14	1M	0,1M	Sigma	SLBS7651	Flavor enhancer, flavoring agent or adjuvant, pH control agent, synergist
Pyruvic acid	C3H4O3	80,06	100,00	0,06	1M	0,1M	Daejung	P3470SG1	Flavoring agent or adjuvant
Betaine	C5H11NO2	117,15	98,00	0,12	1M	0,1M	Daejung	B1549PE1	Flavoring agent or adjuvant
Taurine	C2H7NO3S	125,15	99,00	0,13	1M	0,1M	Sigma	12515DU	Flavoring agent or adjuvant
Riboflavin	C17H20N4O6	376,37	100,00	0,19	1M	0,1M	Sigma	069k1585	Color or coloring adjunct, flavor enhancer, flavoring agent or adjuvant, nutrient supplement
Thiamine hydrochloride	C12H17ClN4OS.HCl	337,27	98,00	0,17	1M	0,1M	Daejung	T0027QL1	Flavoring agent or adjuvant, nutrient supplement
L- Ascorbic acid	C6H8O6	176,12	99,00	0,18	1M	0,1M	TCI	KNBGE	Antimicrobial agent, antioxidant, color or coloring adjunct, dough strengthener, flavor enhancer, flavoring agent or adjuvant, nutrient supplement, pH control agent, sequestrant, stabilizer or thickener

Table S2. Main components declared on selected commercial probiotics in South Korea and the Philippines.

Product type	Declared microorganisms*	Country of origin	Formulations / ingredients
Tablet	**L. reuteri DSM 17938*	Sweden	Isomaltose, xylitol, palm oil, calcium stearate, strawberry flavor, citric acid.
Soft gel capsule	*B. breve subsp. breve* M-16v<br>*B. infantis subsp. infantis* M-63<br>*B. longum* BB536<br>*E. faecalis* TH10<br>*L. acidophilus* ATCC SD5212<br>*L. brevis* NBRC 3345<br>*L. bulgaricus* NBRC 13953<br>*Lc. casei* TO-A<br>*L. fermentum* NBRC 3071<br>*L. helveticus subsp. jugurti* BBRC 3809<br>*Lp. plantarum* TO-A<br>*S. thermophilus* NBRC1397	Japan	Oligosaccharides, amino acids, vitamin B6, B12, B3, niacinamide, folic acid, pantothenic acid, biotin, inositol, riboflavin, iron, potassium, phosphorus, sodium
Powder	*Lc. rhamnosus GG* and *PXN 54*	Denmark	Mannitol, inulin, silicon dioxide
Powder	*Lc. casei PXN 37*<br>*S. thermophilus* PXN 66<br>*B. breve* PXN 25<br>*L. acidophilus PXN 35*<br>*B. infantis* (child specific) PXN 27<br>*L. bulgaricus PXN 39*	United Kingdom	Fructo-oligosaccharides (FOS)
Powder	*L. acidophilus*<br>*B. longum*<br>*B. bifidum* BGN4	South Korea	Vegetable cream, FOS, yogurt powder flavor
Powder	*B. longum* BORI<br>*L. acidophilus* A031<br>*E. faecium* BH06	South Korea	FOS, maltose, galacto-oligosaccharide, corn powder, seaweed calcium, vitamin-mineral mix, aloe.
Gummy candies	*Lc. lactis*<br>*S. thermophilus*<br>*E. faecium*<br>*L. plantarum*	South Korea	Sucrose, starch syrup, D-Sorbitol, gelatin, yogurt flavor, citric acid, titanium dioxide, pectin, coating oil, carnauba cax, soybean phospholipids trisodium citrate, skim milk, stevia

* The nomenclature of the former genus *Lactobacillus* has been retained in the Table as given in the product labels. Full information on the new genera can be obtained from: Zheng et al. (2020). A taxonomic note on the genus *Lactobacillus*: Description of 23 novel genera, emended description of the genus *Lactobacillus* Beijerinck 1901, and union of *Lactobacillaceae* and *Leuconostocaceae*. *Int. J. System. Evol. Microbiol.* **70**: 2782-2858. DOI 10.1099/ijsem.0.004107.
